# Strangulated Epiploic Appendagitis in a Spigelian Hernia: A Rare Clinical Encounter

**DOI:** 10.7759/cureus.110385

**Published:** 2026-06-07

**Authors:** Mohmmad S Hassan, Moksha Ravi Dadlani, Mira Harb, Salwa Asif, Jaiger Chinthamani

**Affiliations:** 1 Radiology, Thumbay University Hospital, Ajman, ARE; 2 Surgery, Thumbay University Hospital, Ajman, ARE

**Keywords:** abdominal wall hernia, computed tomography, epiploic appendagitis, hernia strangulation, spigelian hernia

## Abstract

A Spigelian hernia (SH) is an uncommon abdominal wall hernia occurring along the semilunar line and is often difficult to diagnose due to its intermuscular location. The presence of an epiploic appendage within the hernial sac is extremely rare and may further complicate diagnosis, especially when associated with inflammation or strangulation. We report a case of a 37-year-old female presenting with persistent left lower quadrant abdominal pain, fever, and nausea. Clinical examination revealed localized tenderness, while laboratory findings demonstrated leukocytosis and elevated inflammatory markers. Ultrasound of the abdomen was inconclusive. Computed tomography (CT) demonstrated a fat-attenuating lesion with a hyperattenuating rim and surrounding inflammatory changes, herniating through the semilunar line, consistent with the diagnosis of epiploic appendagitis (EA) within an SH, which is a rare condition with an incidence of 8.8 per million patients. The patient underwent successful laparoscopic reduction and repair with an uneventful recovery.

## Introduction

A Spigelian hernia (SH) is an uncommon abdominal wall hernia that occurs through a defect in the spigelian fascia along the semilunar line, most frequently in the lower abdomen [1,2]. Its intermuscular location often makes clinical diagnosis challenging, as it may not present with a visible swelling or palpable mass [1,3]. Predisposing factors include conditions that increase intra-abdominal pressure, such as obesity and weakening of the abdominal wall musculature [2,3]. Despite its rarity, SH is clinically significant due to its relatively high risk of incarceration and strangulation, often necessitating surgical intervention once identified [2,4]. Epiploic appendagitis (EA) is an extremely rare condition with an incidence of 8.8 per million patients [5]. It is usually a self-limiting inflammatory condition caused by torsion or vascular compromise of epiploic appendages, and may mimic other causes of acute abdomen [6].

Strangulated EA within an SH is an exceedingly rare condition with only a limited number of cases reported in the literature [3,5,7]. This unusual combination presents a diagnostic challenge due to nonspecific clinical features and may alter the clinical course. We report a case of EA occurring within an SH, highlighting the role of imaging in diagnosis and management.

## Case presentation

A 37-year-old female, with a history of hypothyroidism and a body mass index (BMI) of 36.91 kg/m^2^, presented with a 12-day history of progressively worsening localized left lower abdominal pain, accompanied by fever and nausea. There was no history of vomiting, lower urinary tract symptoms, bowel habit changes, or prior similar episodes. On examination, the patient was febrile and had localized tenderness in the left iliac fossa. No visible swelling or palpable abdominal wall mass was appreciated. Laboratory investigations revealed leukocytosis (white blood cell count 14.9 × 10³/µL, reference range: 4.0-10.0 × 10³/µL) and elevated inflammatory markers, including C-reactive protein (CRP) of 22 mg/L.

An initial abdominal ultrasound revealed inflammatory changes within the anterior abdominal muscular plane on the left iliac fossa (Figure 1).

**Figure 1:**
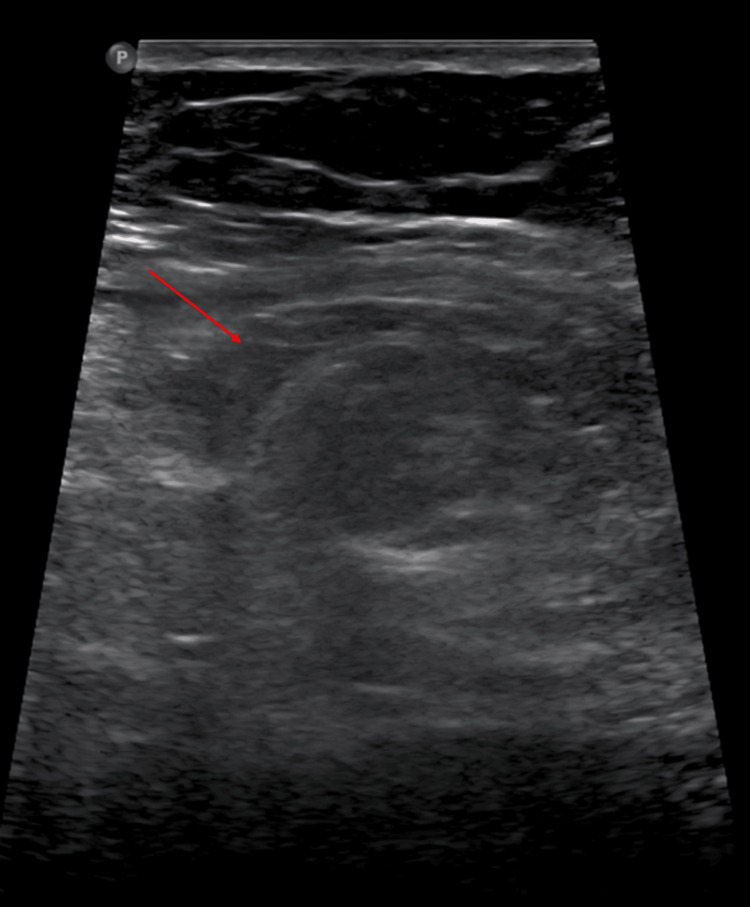
Ultrasound abdomen A hypoechoic lesion with an oval shape (red arrow), measuring 2 × 1.5 cm, was detected within the anterior muscular plane, demonstrating avascularity and accompanied by surrounding inflammatory changes.

To further characterize the inflammatory mass, a non-contrast CT scan of the abdomen was performed, which demonstrated a well-defined oval, fat-attenuating lesion measuring approximately 2 × 1.8 × 1.8 cm, surrounded by inflammatory fat stranding and a thin hyperattenuating rim (Figure 2). A contrast-enhanced CT scan is preferred in such cases to demonstrate ischemic changes. Contrast CT was deferred in our patient as there was a history of allergic reaction to CT contrast.

**Figure 2:**
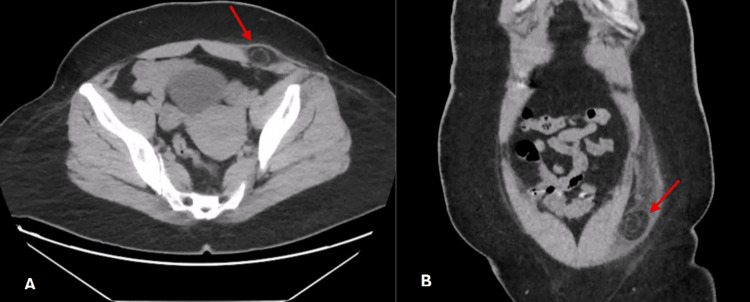
CT abdomen CT images demonstrating an ovoid fat-attenuating lesion (red arrow) with surrounding inflammatory stranding within the muscular layer of the left anterior abdominal wall, consistent with epiploic appendagitis within a Spigelian hernia. (A) Axial CT image showing the lesion embedded along the semilunar line. (B) Coronal CT image showing the extent of the lesion.

The lesion was located adjacent to the sigmoid colon and was seen herniating through a defect measuring 14 x 13 mm along the semilunar line into the intermuscular plane (Figure 3).

**Figure 3:**
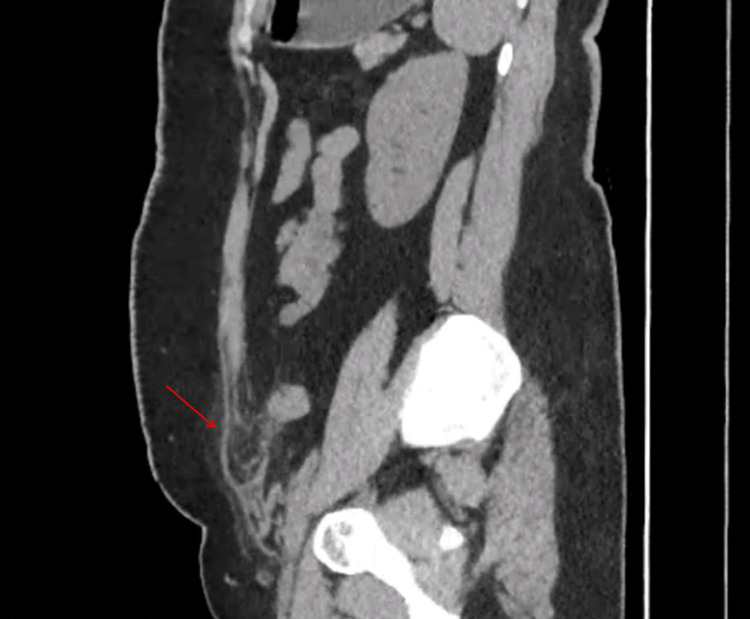
CT imaging: sagittal Sagittal CT image demonstrating an ovoid fat-attenuating lesion (red arrow) with surrounding inflammatory stranding in close proximity to the sigmoid colon, extending into the anterior abdominal wall, consistent with epiploic appendagitis within a Spigelian hernia. Minimal free fluid noted around the herniated sac.

These findings were consistent with EA within an SH, with features suggestive of strangulation (Figure 4).

**Figure 4:**
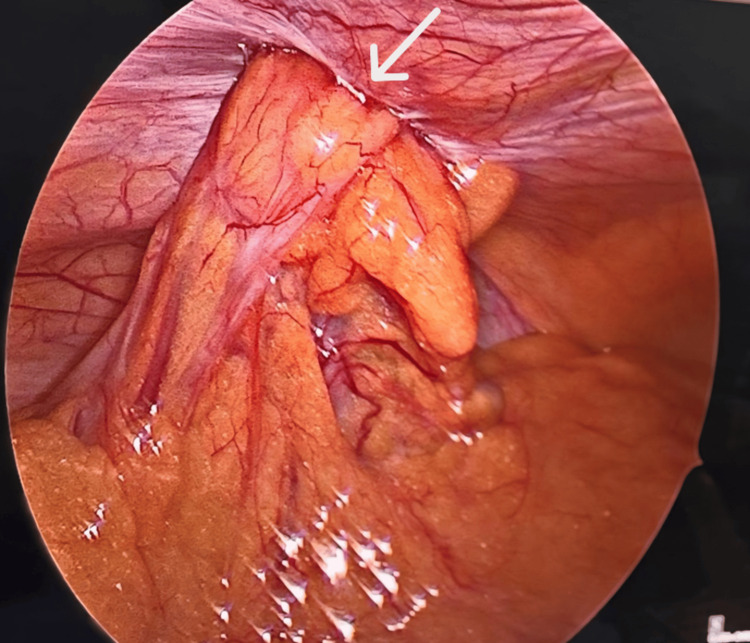
Intraoperative view Intraoperative image demonstrating a colonic epiploic appendage herniating through a defect in the anterior abdominal wall (white arrow).

The patient subsequently underwent laparoscopic surgery. CT findings of EA within an SH were confirmed and repaired. No evidence of bowel ischemia or perforation was noted. The EA was viable and not resected. The postoperative course was uneventful, and the patient was discharged in stable condition. No collection or inflammation was noted at the operative site in the follow-up ultrasound.

## Discussion

SH accounts for approximately 0.1-2% of all abdominal wall hernias and typically occurs through a defect in the spigelian fascia adjacent to the semilunar line. It demonstrates a female preponderance and is associated with conditions that increase intra-abdominal pressure, such as obesity, pregnancy, and chronic straining [1-4].

Although preperitoneal fat, omentum, and loops of the small intestine are the typical contents of an SH, the occurrence of an epiploic appendage, as noted in our patient, is uncommon and has been reported rarely [3,4,8-10]. A notable feature of SH is its relatively high risk of incarceration and strangulation, which may, albeit rarely, involve the epiploic appendage [3,5,7].

Epiploic appendagitis is an uncommon and typically self-limiting cause of acute abdominal pain; however, when it occurs within a hernia sac, it may result in persistent symptoms and an increased risk of complications, including strangulation, thereby necessitating surgical management [2,4,5,7]. The present case highlights this rare association.

A review of the limited literature (Table 1) underscores the rarity of this entity, with only a handful of reported cases over the past decade [5,7,10,11].

**Table 1:** Review of the literature M: male, F: female, CT: computed tomography, LLQ: left lower quadrant

	Year of publication	Authors	Age of patient/gender	Clinical examination	Laterality	Ultrasound	CT	Management
1	2010	Coulier B et al. [11]	49/F	“Olive-shaped” mass palpable	Left	18 x 10 mm hyperechoic avascular mass	Yes	Laparoscopic repair
2	2011	Capaccio E [10]	84/M	Small palpable tender mass	Left	5 cm mass. Hyperechoic border, hypoechoic internal rim + heterogenetic central mass	Yes	Surgery – open repair
3	2021	Chióng Neto FV et al. [7]	82/F	No palpable mass	Left	NA	Yes	Exploratory laparotomy
4	2023	Lima DL et al. [5]	52/M	Irreducible LLQ mass, tender to palpation	Left	NA	Yes	Robotic-assisted surgical approach
5	2026	Hassan MS et al. (present study)	37/F	No palpable mass	Left	Inconclusive	Yes	Laparoscopic reduction

Most patients were middle-aged to elderly, with a slight female predominance. Clinically, a palpable mass was inconsistently present, reflecting the intermuscular location of SH; notably, two cases, including the present one, had no detectable mass on examination [7]. All reported cases involved the left side, suggesting a possible predilection for the left lower abdominal wall.

Imaging findings across reported cases emphasize the pivotal role of CT in diagnosis [5,7,10,11]. Ultrasound findings were variable, ranging from a clearly demonstrated hyperechoic avascular mass to inconclusive results; CT consistently identified both the hernial defect and the inflamed epiploic appendage [10,11]. The characteristic CT features of epiploic appendagitis, including a well-defined fat-density lesion with a hyperattenuating rim and surrounding inflammatory stranding, allow reliable differentiation from other causes of acute abdomen [3,6]. In the present case, ultrasound was inconclusive, whereas CT accurately established the diagnosis, in concordance with prior reports.

Management in all reported cases was surgical, although the approach has evolved over time from open repair and exploratory laparotomy to minimally invasive laparoscopic and robotic-assisted techniques [5,7,10,11]. The current case was successfully managed laparoscopically, reflecting contemporary trends toward minimally invasive intervention.

The intermuscular location of SH contributes to diagnostic difficulty and underscores the importance of imaging in patients with atypical or unexplained abdominal pain [3]. This case, in conjunction with prior reports, highlights the need for a high index of suspicion. While uncomplicated epiploic appendagitis is typically treated conservatively, prompt diagnosis via CT is crucial in instances linked to SH, where surgical intervention might be necessary due to incarceration or strangulation.

## Conclusions

This case highlights an uncommon presentation of EA occurring within an SH, a combination that can pose significant diagnostic difficulty due to its nonspecific clinical features and overlap with more common causes of acute abdominal pain. The intermuscular location of SH further obscures clinical detection, often necessitating a high index of suspicion. In this context, CT plays a pivotal role in accurately identifying both the hernia defect and its contents, allowing for timely diagnosis and appropriate surgical planning.

Early recognition of this rare entity is essential to prevent complications such as incarceration and strangulation. Awareness of the possibility of atypical hernia contents, including epiploic appendages, can enhance diagnostic accuracy and reduce delays in management. Occult SH with unusual contents should be considered in persistent localized abdominal pain, and CT is essential for diagnosis.
